# Supplementary material for: MeioBIOME: A snakemake workflow for the parallel analysis of meiofaunal genomes and host-associated bacteria/archaea
Source: bioRxiv. 2026 Jul 24:2026.07.23.740139. Preprint. [Version 1] doi: 10.64898/2026.07.23.740139 (PMC13419816; doi:10.64898/2026.07.23.740139)
Supplement: 1 [file NIHPP2026.07.23.740139v1-supplement-1.pdf]

bioRxiv preprint doi: https://doi.org/10.64898/2026.07.23.740139; this version posted July 24, 2026. The copyright holder for this preprint (which was not certified by peer review) is the author/funder, who has granted bioRxiv a license to display the preprint in perpetuity. It is made available under a CC-BY 4.0 International license.

Supplementary Figures

Figure S1. Directory structure and Snakemake rulegraph of MeioBIOME. The A) organization of the snakemake directory, which includes workflow rules, Conda environments,

bioRxiv preprint doi: https://doi.org/10.64898/2026.07.23.740139; this version posted July 24, 2026. The copyright holder for this preprint (which was not certified by peer review) is the author/funder, who has granted bioRxiv a license to display the preprint in perpetuity. It is made available under a CC-BY 4.0 International license.

scripts, and config files, B) structure of the final directory output, and C) Snakemake rulegraph including all the internal subcommands (**Supplementary Table AA**).

Supplementary Tables

Table S1. List of all the open-source tools and dependencies implemented in MeioBIOME. A comprehensive list of the internal subcommands and open-sourced software that are implemented in the Snakemake pipeline. We encourage users to cite the appropriate tools.

Table S2. Metadata of the publicly available single-worm metagenomic datasets and summary of the host genes recovered. Nematodes belonging to four nematode families (Thoracostomopsidae, Oncholaimidae, Sphaerolaimidae, and Leptosomatidae) were isolated from four distinct habitats (Tybee Island, Antarctica, Bodega Bay, and Dolphin's Reef, Florida). The 18S rRNA was recovered using barrnap, and the 18S rRNA gene was BLAST to verify the taxonomic identification of the host. BUSCOs were recovered using the Nematoda-specific OrthoDB v12. The total MAGs include high, medium, and low-quality MAGs.

Table S3. Summary of the nematode-associated bacterial MAGs recovered using MeioBIOME. The colors indicate whether the metagenome-assembled genomes (MAGs) were classified as high (green), medium (blue), or low (light red) quality according the CheckM2. The two contaminant MAGs are highlighted dark red. MAGs were classified using the GTDB-Tk workflow.
